# Introductory Lecture

**Published:** 1846-01-01

**Authors:** Walter Channing


*Introductory Lecture, by Walter Channing, M. D.*—The Boston Medical and Surgical Journal has published the annual address introductory to the lectures at the Boston School of Medicine, which was given, this year, by Walter Channing, M. D., Professor of Midwifery and Medical Jurisprudence. The subject is "*The Medical profession and its preparation.*" We have perused it with much gratification. Prof. Channing is a chaste and classical writer, and we think that this address, if published in a pamphlet form, and extensively circulated, will do good. Its spirit, and, in general, the views which it sets forth are calculated to elevate and infuse vigor into the medical profession. We must beg leave, however, respectfully to join issue with the talented writer on one point. He seems to attribute much of the depreciation of the medical profession in public sentiment to the popular study of Anatomy and Physiology. We cannot but think this is an erroneous opinion, and one which it would be injurious to inculcate. That a superficial study of these sciences, in some instances, induces a kind of self-conceit, which arrogates an all-sufficient degree of medical knowledge, is doubtless true; time and experience, however, correct these absurdities. In the main, if these studies tend to bring the profession into low esteem, we are bound, as it seems to us, to attribute it to the incompetency of a large class in the profession to represent the actual condition of medicine. Here lies, as we regard it, the secret of the depreciation of our profession. The standard of medical education is too low, and access into the ranks of the profession obtained upon too easy terms. Let the profession be composed of members who have submitted to the preparation and discipline which Prof. Channing advises in another portion of his discourse, and the more the public become familiar with knowledge pertaining to the human constitution, the better the profession will be appreciated. As it is, if a reform in medical education cannot be effected, general diffusion of Physiological knowledge would seem to be necessary for a just discrimination by the public of medical qualifications. We admit that it is not fully competent to this end, but it is approximatively, and may be of considerable utility until a diploma, or license has more virtue than at present. In perusing Prof. Channing's discourse, we have the advantage of many of our readers, in the recollection of his agreeable and impressive manner of delivery—as it was some fifteen years ago, and as we presume it is still. We trust his efforts to inculcate sonnd knowledge, and to inspire with dignified and worthy sentiments those who have the benefit of his instructions, may be continued for many years to come.

